# Lack of Middle East Respiratory Syndrome Coronavirus Transmission from Infected Camels

**DOI:** 10.3201/eid2104.141949

**Published:** 2015-04

**Authors:** Maged G. Hemida, Abdulmohsen Al-Naeem, Ranawaka A.P.M. Perera, Alex W.H. Chin, Leo L.M. Poon, Malik Peiris

**Affiliations:** Kafrelsheikh University, Egypt (M.G. Hemida); King Faisal University, Hofuf, Saudi Arabia (M.G. Hemida, A. Al-Naeem);; The University of Hong Kong, Hong Kong, China (R.A.P.M. Perera, A.W.H. Chin, L.L.M. Poon, M. Peiris)

**Keywords:** zoonoses, Middle East respiratory syndrome, MERS, genomics, phylogeny, mutation, transmission, dromedary, camel, Saudi Arabia, viruses

## Abstract

To determine risk for Middle East respiratory syndrome coronavirus transmission from camels to humans, we tested serum from 191 persons with various levels of exposure to an infected dromedary herd. We found no serologic evidence of human infection, suggesting that zoonotic transmission of this virus from dromedaries is rare.

Cases of Middle East respiratory syndrome (MERS) in humans continue to be reported from the Arabian Peninsula and the Middle East. The largest number of cases has been reported by Saudia Arabia: 818 cases leading to 351 deaths as of December 5, 2014 (*1*). The causative agent is MERS coronavirus (MERS-CoV), which is endemic to and ubiquitous among dromedary camels in the Arabian Peninsula and East and North Africa; seroprevalence among adult animals is typically >90% (*2*). MERS-CoV infection causes mild upper respiratory illness in dromedaries and remains detectable in nasal swab specimens for ≈1 week (*3*).

To look for serologic evidence of MERS-CoV infection in humans extensively exposed to a herd of infected dromedaries, we assessed seroprevalence among persons in close contact with an infected herd of ≈70 animals in Al Hasa, Saudi Arabia, during peak calving season, December 2013–February 2014 (*4*). The study was approved by the King Faisal University Research Ethics Committee.

## The Study

The dromedaries were maintained in a fenced enclosure with barns. MERS-CoV was first detected in this herd on November 30, 2013; by December 30, of 11 sampled dromedaries, 9 were positive for viral RNA by reverse transcription PCR. The viruses isolated in November and December were genetically identical, suggesting ongoing transmission arising from introduction of a single virus (*4*).

In February 2014, serum samples were obtained from persons with various levels of exposure to camels. Persons were divided into 5 groups.

 Group 1 comprised 4 herdsmen who were in daily contact with the infected herd (feeding, grooming, administering treatment when needed). They frequently consumed fresh unboiled milk from the camels, of which at least 1 dam and 7 calves were retrospectively confirmed to have been MERS-CoV infected (*4*). 

Group 2 comprised 8 persons who had intermittent but regular (several times/week) direct contact with the infected herd (animal management, feeding, manure removal) and included veterinary staff and attendants. Because this herd was also used for veterinary teaching and research, animals were frequently handled for clinical examination and specimen collection. With the exception of disposable gloves, which were worn by the veterinarians during examinations, personal protective equipment (masks, gloves, eye protection) was not used. 

Group 3 comprised 30 veterinary surgeons and clinical support staff working at the Clinical Research Center at the Faculty of Veterinary Medicine and Animal Resources, King Faisal University, Saudi Arabia, who were not exposed to the infected herd. This largest clinical veterinary center in southeastern Saudi Arabia also serves the adjacent countries of the United Arab Emirates, Qatar, Bahrain, Kuwait, and Oman. Camels from across Saudi Arabia and the Gulf states are brought to this research center. Although we did not conduct MERS-CoV testing on camels brought for routine clinical care, some animals may have been shedding MERS-CoV. Staff members came into daily contact with domestic livestock of all species, including dromedaries, of which at least 20 arrived daily for treatment. Disposable gloves were used for examinations, but no respiratory or eye protection was used.

Group 4 comprised 3 workers in a camel abattoir in Al Hasa, where 25–35 camels were slaughtered daily. These workers did not wear personal protective equipment. 

Group 5 comprised 146 persons in the same (Al Hasa) region who were not exposed to camels in their professional work. This group served as negative controls. 

Serum from these 191 persons was tested for MERS-CoV antibodies by using a pseudoparticle neutralization assay that has been previously described, validated, and demonstrated to be at least as sensitive as microneutralization assays (*5*,*6*). Evidence of a >50% reduction of signal was also sought as a more sensitive signal of neutralization, which would have been further confirmed in virus plaque neutralization assays.

Of the 191 human serum samples tested, none had serologic evidence of infection, even when a 50% reduction in luciferase signal was used as the threshold for suspected antibody (including those in groups 1 and 2 who had repeated close contact with the infected herd through December 2013). The virus infecting the herd was genetically similar to viruses that had previously infected humans, including in the amino acid sequence of the virus spike protein (*4*,*7*–*10*). We previously demonstrated that MERS-CoV from this infected herd had the capacity to efficiently infect ex vivo cultures from the human respiratory tract (*7*). These findings imply that group 1 and 2 members were repeatedly exposed over at least 1 month to dromedaries shedding a virus that was potentially competent to infect humans.

## Conclusions

We conclude that MERS-CoV was not highly transmissible from dromedaries to humans with various levels of exposure to this infected dromedary herd. Two other studies have reported lack of serologic evidence of MERS-CoV infection among persons with various levels of exposure to dromedaries (*11*,*12*), but neither study documented MERS-CoV infection in the relevant animals. Studies of MERS-CoV RNA prevalence in nasal swab specimens from dromedaries in the field or abattoirs have found rates of 0%–15% (by PCR) among adults and 35% among calves (1 study) (*4*,*8*–*10*).

A study limitation is that precise events of individual-level human exposure to infected animals cannot be ascertained because different animals were infected at different times over the 1-month period. The timing of serum collection (4–6 weeks after putative exposure) from persons exposed to the infected herd was optimal in terms of detecting a serologic response. However, data on antibody titers after asymptomatic or mild MERS-CoV infections are limited, and our conclusion about lack of human infection must be subject to that caveat. 

Our findings do not imply that dromedaries are not a source of infection for humans. Spillover infection of humans may be more common in other settings in which humans are exposed over sustained periods to animals among which virus prevalence is higher. For example, animals from diverse origins with varying immune status that are housed together in abattoirs before slaughter may provide opportunity for virus amplification and persistence, analogous to that seen with avian influenza virus in live poultry markets (*13*). Our study period (December–February) was timed to coincide with the calving season. However, human cases seem to peak during April–June, although this peak may be skewed by clusters of human-to-human transmission. Future longitudinal studies of dromedaries should be of longer duration.

Our finding that MERS-CoV is poorly transmissible from dromedaries to humans is compatible with the observed epidemiology of MERS-CoV infection in humans, in which human disease is not directly proportional to potential exposure to a virus that seems to be common in dromedary camels. Infections in dromedaries in settings such as abattoirs are regularly documented; thus, the numbers of humans exposed to virus-infected animals must greatly exceed the number of humans with diagnosed MERS-CoV infection. Conversely, some persons seem to acquire infection with apparently minimal or no apparent exposure to camels, even when secondary transmission from other infected humans is excluded. This setting is analogous to that observed for avian influenza (H5N1) virus, in which the virus can be ubiquitous in live poultry markets in some settings but human infection and disease remain stochastic and rare (i.e., not directly proportional to exposure) (*13*). The biological basis for such an epidemiologic pattern remains obscure for both viruses, avian influenza (H5N1) and MERS-CoV, but the heterogeneity of host susceptibility is a hypothesis to be explored (*7*,*14*). Further studies on the mechanisms by which MERS-CoV is transmitted from dromedaries to humans, whether by direct or indirect routes, and the heterogeneity of human susceptibility to this virus are needed.
